# Aberrant expression of human endogenous retrovirus K9-derived elements is associated with better clinical outcome of acute myelocytic leukemia

**DOI:** 10.1186/s12977-025-00661-6

**Published:** 2025-04-01

**Authors:** Ryo Yanagiya, So Nakagawa, Makoto Onizuka, Ai Kotani

**Affiliations:** 1 Department of Hematology and Oncology, Tokai University School of Medicine, Isehara, Japan; 2 Department of Innovative Medical Science, Tokai University School of Medicine, Isehara, Japan; 3 Laboratory of Regulation of Infectious Cancer, Division of Cellular and Molecular Biology, Research Institute for Microbial Diseases, Osaka University, Suita, Japan; 4 Department of Drug Discovery and Biomedical Sciences, Faculty of Medicine, Saga University, Saga, Japan; 5 Department of Molecular Life Science, Tokai University School of Medicine, 143-Shimokasuya, Isehara, Kanagawa 259-1193 Japan; 6 Division of Omics Sciences, Institute of Medical Sciences, Tokai University, Isehara, Japan; 7 Division of Interdisciplinary Merging of Health Research, Micro/Nano Technology Center, Tokai University, Hiratsuka, Japan; 8 Department of Hematological Malignancy, Institute of Medical Science, Tokai University, Isehara, Japan

**Keywords:** Acute myelocytic leukemia, Allogeneic hematopoietic cell transplantation, Endogenous viral elements, Neoantigen, Anti-neoplastic immunity

## Abstract

**Background:**

Acute myelocytic leukemia (AML) is a common hematological malignancy in adults. Although several risk stratifications based on cytogenetic and molecular abnormalities are available to guide the indications for allogeneic hematopoietic cell transplantation (allo-HCT), determining optimal treatment strategies for AML remains challenging. In this study, using transcriptome datasets, we investigated the association between event-free survival (EFS) in intensively treated AML patients and the aberrant expression of endogenous viral element (EVE)-derived open reading frames (ORFs), which have been reported to be associated with the pathophysiology of various malignancies and have the potential to serve as neoantigens in specific cancers.

**Results:**

The expression levels of human endogenous retrovirus family K9 (HERVK9) ORFs were associated with EFS, independent of conventional risk stratification. Furthermore, AML cells with higher levels of HERVK9 expression exhibited enhanced antigen processing and presentation, along with increased expression of genes associated with adaptive immune responses and apoptosis, indicating that aberrant HERVK9 expression may initiate an anti-neoplastic immune response via increased antigen presentation.

**Conclusions:**

HERVK9 expression may have serve as a crucial prognostic indicator that could aid in determining the indications for upfront allo-HCT in AML patients.

**Supplementary Information:**

The online version contains supplementary material available at 10.1186/s12977-025-00661-6.

## Background

Acute myelocytic leukemia (AML) is a common hematological malignancy in adults. Although combination chemotherapeutic regimens of cytarabine plus anthracyclines (so-called “3 + 7-based regimens”) have been established as the gold standard of care for AML for decades [1], some cases remain refractory to cytotoxic agents, necessitating allogeneic hematopoietic cell transplantation (allo-HCT) for treatment [2]. The strong anti-neoplastic effect of allo-HCT mainly consists of two different mechanisms: the cytotoxic effect of high-dose chemoradiotherapy administered before transplantation and the sustained disease control conferred by the alloreactive immune response of donor-derived cells against AML cells after transplantation (the graft-versus-leukemia effect) [3]. Since the latter plays a crucial role in prolonging disease-free survival, various experimental and clinical approaches have been explored to enhance alloreactive anti-neoplastic immune responses [4–7]. Although allo-HCT can achieve deeper remission and prolong disease-free survival, it is also associated with higher treatment-related mortality due to chemotoxicities, severe infection, vaso-occlusive diseases, and alloreactive immune responses against recipient tissues (graft-versus-host disease) [8–12]. Therefore, the decision to perform allo-HCT must be carefully balanced against the potential benefits and risks. Current guidelines recommend upfront allo-HCT (i.e., at the time of initial remission) for newly diagnosed AML cases with a high risk of relapse after chemotherapy [13].

Advances in the understanding of the cytogenetic and genetic abnormalities of AML have led to the stratification of disease outcomes into three risk categories, as reflected in the National Comprehensive Cancer Network 2017 (NCCN 2017) [14] and the 2017 European LeukemiaNet (ELN 2017) [15] guidelines: good (NCCN2017) / favorable (ELN2017), intermediate, and poor (NCCN2017) / adverse (ELN2017). AML cases classified as good/favorable-risk generally respond well to chemotherapy and seldom require allo-HCT at initial remission. In contrast, those classified as poor/adverse-risk typically require allo-HCT for effective disease control. However, the optimal treatment strategy for intermediate-risk AML cases, which includes cases with normal karyotypes and/or the absence of detectable genetic abnormalities, remains controversial. Furthermore, some good/favorable-risk AML cases eventually relapse after completing chemotherapy and require allo-HCT. Therefore, additional classification strategies are required to refine treatment decisions.

Endogenous viral elements (EVEs) are genetic sequences originally derived from viral genes or genomes that have inserted into the germline and transmitted vertically to offspring [16, 17]. In mammals, including humans, the majority of EVEs originate from retroviruses and are classified as endogenous retroviruses (ERVs) because they encode reverse transcriptase and integrase, which facilitate their genomic integration [16]. ERVs belong to the family of long-terminal repeat (LTR) retrotransposons. Aberrant ERV expression has been observed in various malignancies [18, 19], and accumulating evidence suggests that the expression of specific ERV families is linked to cancer pathophysiology. For instance, aberrant expression of human endogenous retrovirus family K (HERVK)-derived sequences has been implicated in the initiation of cancer cell proliferation in solid tumors [20, 21]. Additionally, certain ERVs have been shown to elicit anti-neoplastic adaptive immune responses in cancers, as many retain open reading frames (ORFs) encoding retroviral genes [22]. Importantly, several ERV-derived ORFs can be translated into peptides that function as neoantigens, since they are rarely expressed in normal tissues and contain viral motifs that can serve as antigenic targets [23–25]. These findings suggest that virus-derived peptides may modulate anti-neoplastic immune responses and influence clinical outcomes. However, the role of aberrant EVE expression in hematological malignancies, including AML, remains poorly understood.

We have developed a gEVE database for viral motif-containing EVE-derived ORFs (hereafter called EVE ORFs) encoding over 80 amino acids in 19 mammalian species, including humans [26]. In this study, we utilized the human gEVE data to analyze the aberrant expression of EVE ORFs by examining RNA sequencing (RNA-seq) data obtained from The Cancer Genome Atlas (TCGA) and Sequence Read Archive (SRA) databases. We then statistically evaluated the association between EVE ORF expression and event-free survival (EFS) in AML.

## Methods

### Study approval

Access to the RNA-seq data used in this study (see Supplemental Table S1) was granted by the Institutional Review Board of Tokai University School of Medicine (approval number: 19-R-323).

### Data collection of publicity available RNA-seq data

BAM-formatted RNA-seq data and associated clinical information were obtained for 151 AML cases from TCGA (TCGA-LAML). The BAM data files were converted into FASTQ files using bam2fastq version 1.1.0. Genetic mutation annotations and clinical status at allo-HCT for the 151 cases were extracted from the supplementary information of TCGA-LAML paper [27]. FASTQ-formatted RNA-seq data for 21 AML cases were obtained from the Gene Expression Omnibus (GEO) database (GSE49642). Additionally, FASTQ-formatted RNA-seq data for bone marrow-derived CD34^+^ normal hematopoietic precursor cells (HPCs) were obtained from GEO, with 21 and 8 files from GSE111085 and GSE114922, respectively.

### Differentially-expressed EVE analysis

All FASTQ files were mapped to the human genome (hg38; downloaded from https://hgdownload.soe.ucsc.edu/goldenPath/hg38/bigZips) using HISAT2 version 2.2.1 [28] with default parameters. The reads were then counted and annotated using StringTie version 2.1.6 [29] with the GTF-formatted annotation file of human EVE ORF entries obtained from the gEVE database version 1.1 [26]. To classify EVE ORF families, Repbase annotations [30] that overlapped with gEVE entries were used. DESeq2 version 1.42.0 was used to calculate statistical differences in the EVE expression between AML and normal HPCs, and entries with an adjusted *p-*value < 0.05 were identified as differentially expressed EVEs (DE-EVEs). Gene set enrichment analysis (GSEA) of DE-EVEs was performed based on Repbase EVE family annotations using clusterProfiler version 3.18 [31]. The chromosomal locations of DE-EVEs were visualized using Chromomap version 4.1.1 [32]. All the datasets were accessed on June 10, 2021.

### Differentially-expressed human gene analysis

All FASTQ files were mapped to the human genome using HISAT2 [28] with the “--known-splicesite-infile” option, along with the human gene annotation file: hg38.ncbiRefSeq.gtf downloaded from https://hgdownload.soe.ucsc.edu/goldenPath/hg38/bigZips/genes (accessed on June 10, 2021). StringTie [29] and DESeq2 were used to identify differentially expressed genes (DEGs), with genes meeting an adjusted p-value threshold of < 0.25 and an absolute log2-fold change ≥ 0.5 considered as DEGs. The GSEAs of DEGs were performed using clusterProfiler [31] with three following gene annotation datasets obtained from the molecular signatures database (MSigDB; https://www.gsea-msigdb.org/gsea/msigdb/human/collections.jsp) (accessed on June 10, 2021): Hallmark gene sets, Kyoto Encyclopedia of Genes and Genomes (KEGG) legacy subset of canonical pathways, and Gene Ontology gene sets.

### Cell culture

The cell lines THP1 (RRID: CVCL_0006), KG1 (CVCL_0374), HL60 (CVCL_0002), HEL (CVCL_0001), and K562 (CVCL_0004) were purchased from the Japanese Collection of Research Bioresources. The cells were cultured at 37℃ in a 5% CO_2_ incubator in RPMI-1640 medium (FUJIFILM Wako, #189–02025) supplemented with 10% fetal bovine serum (Gibco, #26140079) and Penicillin-Streptomycin Solution (FUJIFILM Wako, #168-23191).

### Reverse transcription polymerase chain reaction

Total RNA was extracted using Sepasol-RNA I Super G (Nakalai Tesque, #09379-55), following the manufacturer’s protocol. Complementary DNA from RNA with a polyadenylated tail was synthesized using ReverTra Ace qPCR RT Master Mix with gDNA Remover (TOYOBO, #FSQ-301), following the manufacturer’s protocol. Polymerase chain reaction was performed using THUNDERBIRD SYBR qPCR Mix (TOYOBO, #QPS-201) and StepOnePlus Real-Time PCR System (Applied Biosystems). The primer sequences are shown in Supplemental Table S2.

### Statistical analysis

All statistical analyses were performed in R version 4.2.2 for Windows. Principal component analysis (PCA) was performed using the prcomp function in the base R package. The Mann-Whitney U test was used for comparison of transcripts per million (TPM) values between RNA-seq datasets, with a significance threshold of *p*-value < 0.05. Uniform manifold approximation and projection (UMAP) were performed using Umap version 0.2.10.0. The optimal cutoff value for grouping in survival analysis was determined using Maxstat version 0.7.25, with a Hosmer-Lemeshow test adjusted *p*-value < 0.1 considered significant. Differences in the event-free survival (EFS) periods between groups were analyzed using the log-rank test. Multivariate analysis of factors affecting EFS was conducted using the Cox proportional hazards model. Fisher’s exact test was used to compare binary categorical subgroups.

## Results

### Validation of analyzed datasets

First, the quality of the RNA-seq datasets used in this study was examined: patient-derived AML cells (151 and 21 samples from TCGA-LAML and GSE49642, respectively) and CD34^+^ normal HPCs derived from bone marrow of healthy volunteers (23 and eight samples from GSE111085 and GSE114922, respectively). The RNA-seq reads of each sample were mapped to the human genome, transcripts per million (TPM) values for gEVE entries were obtained, and PCA was performed (see Materials and Methods). The AML patient-derived datasets and HPC-derived healthy control datasets were distinctly separated by PC1 (contribution ratio: 23%; Supplemental Figure S1a-b). Batch effects between individual datasets were observed in PC2, although the contribution ratio was relatively low (7%). Furthermore, UMAP using PCs with a cumulative proportion exceeding 80% (PC1 to PC67) clearly distinguished AML from HPC samples (Supplemental Figure S1c).

We further assessed the quality of the AML cell-derived samples using *CD34* TPM values, as CD34-positive cell extraction was performed during sample preparation for the healthy control datasets (i.e., GSE111085 and GSE114922). The TPM value distribution of the TCGA-LAML dataset did not significantly differ from those of GSE111085 and GSE114922 datasets, whereas GSE49642 dataset exhibited significantly lower TPM values (Supplemental Figure S1d). These results indicate that contamination by non-leukemic cells was a greater concern in the GSE49642-derived samples. Thus, TCGA-LAML was used as the primary dataset, with GSE49642 serving as a validation dataset.

### Detection of differentially expressed EVE ORFs

**Fig. 1:**
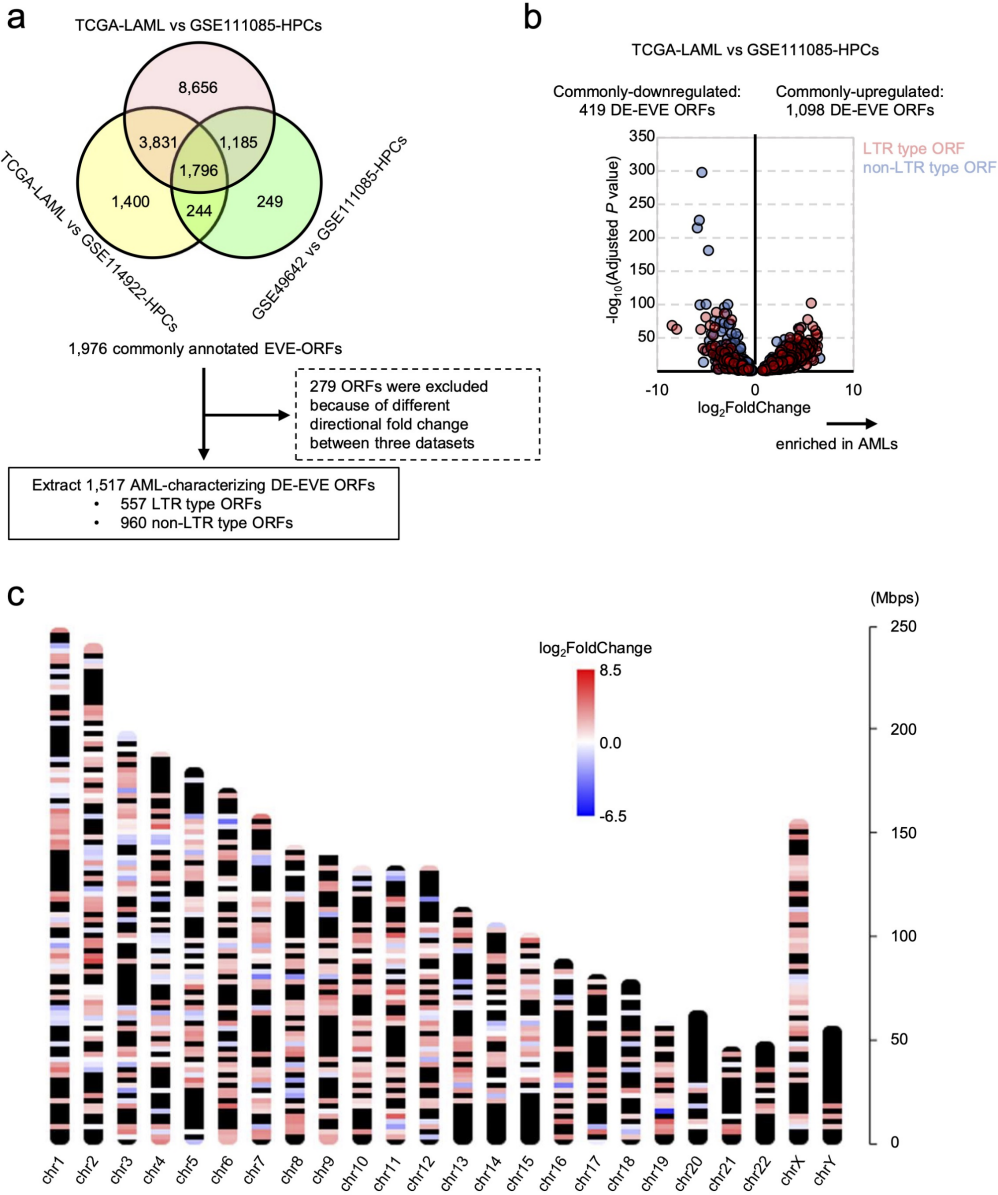
Identification of differentially expressed gEVE entries (DE-EVE ORFs) in AML cell-derived transcripts using multiple transcriptome datasets. **(a)** Schematic representation of DE-EVE ORF detection using transcriptome data from AML cells (TCGA-LAML and GSE49642) and normal HPCs (GSE111085 and GSE114922). **(b)** Volcano plot of 1,517 DE-EVE ORFs in AML cells from TCGA-LAML compared with those in HPCs from GSE111085. **(c)** Chromosomal localization of 1,517 DE-EVE ORFs. The color scale indicates log_2_-fold change in DE-EVE ORF expression in AML cells from TCGA-LAML compared to those in HPCs from GSE111085

We conducted differential expression (DE) analysis of gEVE entries (hereafter called “DE-EVE ORFs”) across three independent tests (TCGA-LAML vs. GSE111085, TCGA-LAML vs. GSE114922, and GSE49642 vs. GSE111085). A total of 1,796 DE-EVE ORFs were commonly identified across these analyses (Fig. 1a and Supplemental Table S3-S5). To minimize batch effects between datasets rather than reflecting true difference between AML and HPCs, 279 DE-EVE ORFs with inconsistent directional fold changes across the three analyses were excluded. As a result, 1,517 DE-EVE ORFs were classified as AML-characterizing DE-EVE ORFs, including 557 LTR-type and 960 non-LTR-type EVE ORFs (Fig. 1a-b and Supplemental Table S6).

PCA of the TPM values for the 1,517 DE-EVE ORFs in TCGA-LAML samples revealed that the cumulative proportion for 72 PCs exceeded 80% (80.03%; Supplemental Figure S2a). UMAP of these PCs suggests that DE-EVE ORF expression patterns in AML were independent of common cytogenetic abnormalities and morphological features (referring to the French-American-British [FAB] classification), with the exception of FAB-M3 (acute promyelocytic leukemia; equal to the cytogenic class of *PML*::*RARA*), which is a well-known prognostic factor in AML [33, 34] (Supplemental Figure S2b-c).

The genomic loci of the 1,517 DE-EVE ORFs were mapped to human chromosomes, and genome-wide hotspots of DE-EVE ORF expression were visualized (Fig. 1c). Notably, chromosome 19 exhibited the highest density of DE-EVE ORF expression sites, consistent with previous reports identifying it as an enriched site for LTR retrotransposons [35–38].

### Enrichment analysis of DE-EVE families

**Fig. 2:**
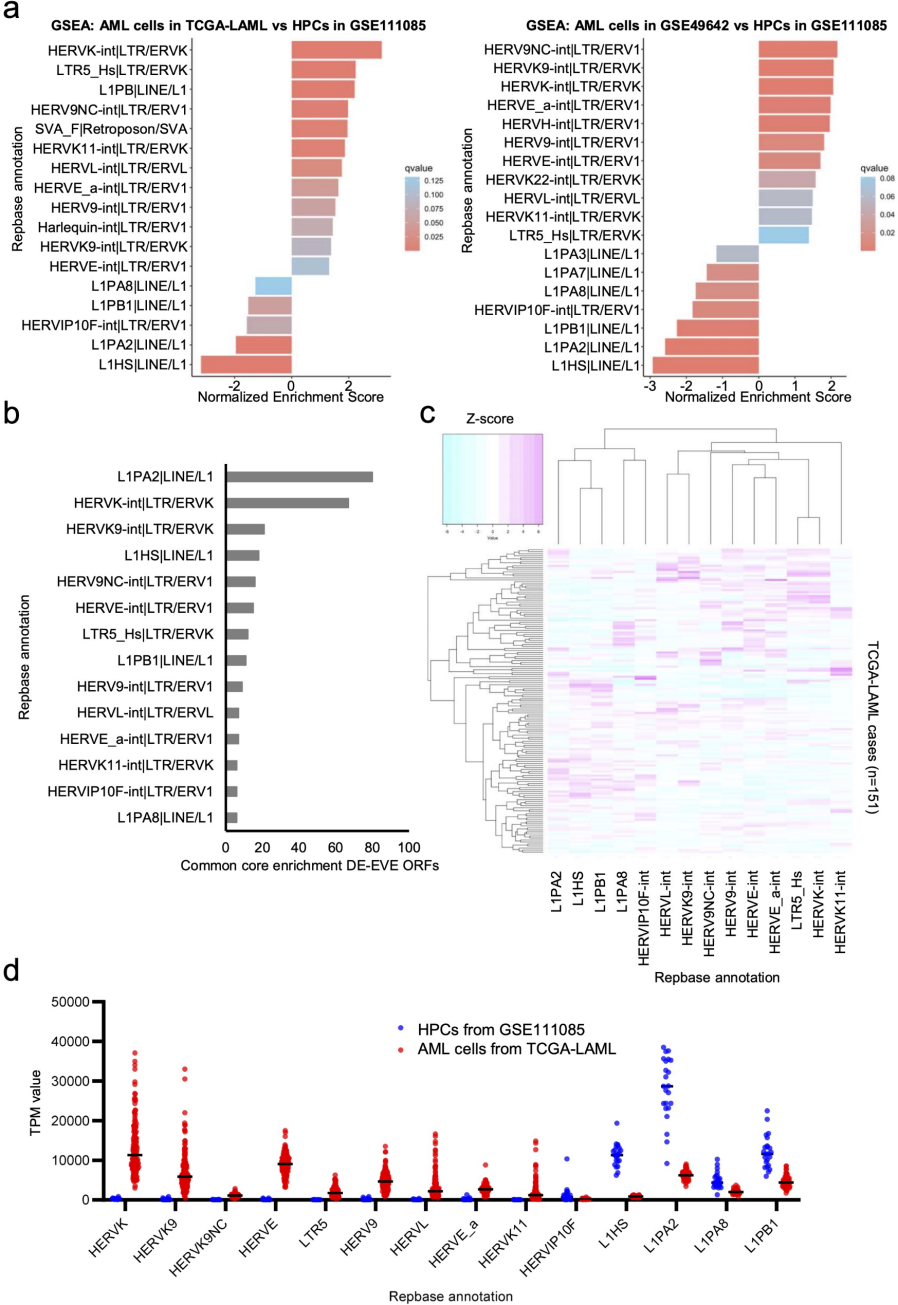
Identification of DE-EVE families using gene set enrichment analysis (GSEA). **(a)** GSEA bar plot of 1,517 DE-EVE ORFs, annotated using the GMT-formatted annotation file of Repbase-EVE families. **(b)** Common DE-EVE families and common core enriched DE-EVE ORFs in two analyzed datasets described in **a**. **(c)** Expression profile heatmap of 14 extracted DE-EVE families in AML cells from TCGA-LAML. **(d)** Expression value (transcripts per million; TPM) of DE-EVE families in AML cells from TCGA-LAML (red) and HPCs in GSE111085 (blue). Bars indicate the median

EVE families differentially expressed in AML cells were identified from two paired comparisons (TCGA-LAML AML cells vs. GSE111085 HPCs and GSE49642 AML cells vs. GSE111085 HPCs), using Repbase annotation obtained from the gEVE database (see Materials and Methods). As a result, 14 EVE families (10 LTR types and four LINE types) were commonly identified as DE-EVE families, containing a total of 355 core-enrichment DE-EVE ORFs (Fig. 2a-b and Supplemental Table S7-S9). Among the 14 highly expressed EVE families, expression patterns in AML cells varied between LTR-type and LINE-type elements (Fig. 2c). The HERVK and HERVK9 families exhibited broader expression distributions than other families, suggesting high heterogeneity in the expression across AML patients (Fig. 2d).

### Correlation of HERVK9 expression with EFS of AML cases with 3 + 7-based intensive chemotherapy

**Fig. 3:**
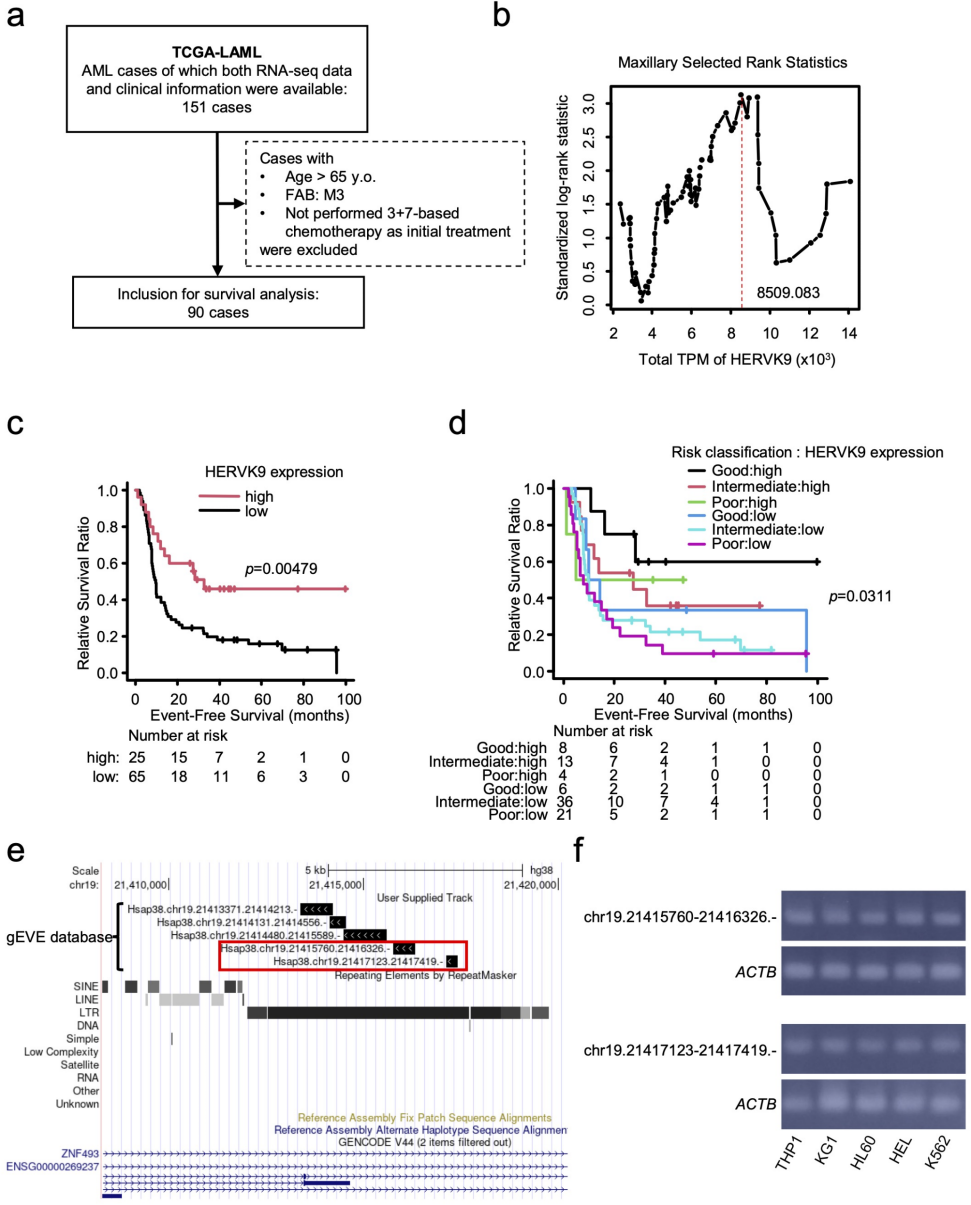
Association between HERVK9 expression and prognosis of AML cases with 3 + 7-based intensive chemotherapy. **(a)** Case selection flowchart for survival analyses. Detailed patient information from TCGA-LAML is available in Supplemental Table S10. **(b)** Determination of the optimal cutoff value for HERVK9-derived ORF expression for log-rank analysis using maxillary selected rank statistics in 90 AML cases. **(c)** EFS curves of AML cases stratified by HERVK9 expression levels. **(d)** EFS curves of AML cases stratified by both HERVK9 expression levels and NCCN2017 risk classification. **(e)** Genomic localization of HERVK9-derived ORFs on chromosome 19 (red square). **(f)** Reverse transcription polymerase chain reaction analyses of two annotated DE-EVEs shown in **(e)***ACTB* was used as an internal control

As previously shown, the expression profiles of DE-EVEs were independent of known cytogenetic risk factors, except for *PML*::*RARA* (FAB-M3; Supplemental FigureS1b-c). To identify AML-related EVE family candidates, we analyzed the correlation between the DE-EVE family expression levels and prognosis in AML cases. Among the 151 AML cases with clinical information from TCGA-LAML, we excluded the following cases to minimize intervention-derived noise in survival data analysis (Fig. 3a): [1] elderly cases (> 65 years old), as they were generally considered “allo-HCT ineligible” at that time and received different treatment strategies compared to allo-HCT-eligible cases; [2] FAB-M3 cases, as their treatment strategy was completely different from that of the others; and [3] cases that received non-intensive treatments (i.e., other than 3 + 7-based regimens) as an initial treatment. After these exclusions, a sub-cohort of 90 cases remained for the survival analyses (Table 1 and Supplemental Table S10). Among the 14 highly expressed DE-EVE families, only the HERVK9 family expression value correlated with EFS using a maxillary selected ranked analysis, with a TPM cutoff of 8,509.1 (Fig. 3b and Supplemental Figure S3). Kaplan-Meier survival analysis of the 90 AML cases, stratified by HERVK9 expression values with the measured cutoff, revealed that higher HERVK9 expression was associated with prolonged EFS (Fig. 3c). This result was validated in cases that did not undergo upfront allo-HCT (Supplemental Figure S4a). Notably, this tendency was also observed in AML cases classified as good- and intermediate-risk per NCCN2017 guidelines (Fig. 3d and Supplemental Figure S4b). Multivariate analysis using the Cox proportional hazards model was applied to the 90 AML cases, incorporating previously known prognostic factors (age, FAB classification, NCCN2017 molecular risk stratification, and gene mutations specifically defined in ELN2017) along with HERVK9 expression status to assess its impact on prognosis. The results indicated that HERVK9 expression status serves a risk factor independent of previously known factors (Table 2).

**Table 1:** Patient characteristics of 90 AML cases from TCGA-LAML for survival analyses

Factor		
Age (range)	48.5 (21–65)	
Sex, n		
Male	49 (54.4%)	
Female	41 (45.6%)	
FAB classification, n		
M0	8 (8.9%)	
M1	27 (30.0%)	
M2	25 (27.8%)	
M4	19 (21.1%)	
M5	10 (11.1%)	
M6	1 (1.1%)	
Cytogenetic abnormality, n		
Normal karyotype	43 (47.8%)	
*RUNX1*::*RUNX1T1*	6 (6.7%)	
*CBFB*::*MYH11*	8 (8.9%)	
*MLL* translocation, t(9;11)	1 (1.1%)	
Other intermediate risk cytogenetic abnormality	9 (10.0%)	
*MLL* translocation, otherwise	4 (4.4%)	
*BCR*::*ABL1*	2 (2.2%)	
Other poor risk cytogenetic abnormality	7 (7.8%)	
Complex cytogenetics	8 (8.9%)	
Data not available	2 (2.2%)	
NCCN2017 risk stratification, n		
Good	14 (15.6%)	
Intermediate	49 (54.4%)	
Poor	25 (27.8%)	
Data not available	2 (2.2%)	
Upfront allo-HCT, n		
Performed	33 (36.7%)	
Not performed	57 (63.3%)	
	Total *n* = 90	

*Abbreviations* FAB, French-American-British; NCCN, National Comprehensive Cancer Network; allo-HCT, allogeneic hematopoietic stem cell transplantation

**Table 2:** Cox proportional hazards model for EFS of 90 AML cases

Factor	Hazard ratio	95%CI	*P*-value
Age	1.010	0.990–1.031	0.32440
FAB (vs. M0)			
M1	2.077	0.732–5.892	0.16940
M2	1.471	0.512–4.226	0.47390
M4	2.642	0.929–7.519	0.06858
M5	2.227	0.572–8.674	0.24840
M6	2.251	0.158–32.04	0.54940
NCCN2017 risk stratification (vs. good)			
Intermediate	1.493	0.608–3.667	0.38230
Poor	2.045	0.762–5.490	0.15560
Gene mutations contributing to risk stratifications			
*ASXL1*	0.717	0.6633-7.760	0.78460
*TP53*	2.142	0.4532–10.120	0.33650
*RUNX1*	2.161	0.8089–5.775	0.12430
HERVK9 expression (vs. high expression)			
Low expression	2.615	1.166–5.866	0.01971

*Abbreviations* EFS, event-free survival; CI, confidence interval; HERVK9, human endogenous retrovirus K9

Next, we analyzed the associations between HERVK9 expression status and the cytogenetic and molecular abnormalities described in the NCCN2017 and/or ELN2017 guidelines, using all 151 AML cases from TCGA-LAML (Supplemental Table S10). While chromosomal translocations associated with core-binding factors (i.e., *RUNX1*::*RUNX1T1* and *CBFB*::*MYH11*) were associated with higher HERVK9 expression, other cytogenetic and molecular abnormalities did not show a significant relationship with HERVK9 expression (Supplemental Table S11).

We further investigated the genomic locations of HERVK9 associated with AML prognosis. Among the 21 HERVK9-derived ORFs that are commonly enriched (Supplemental Table S7-S9), two located on chromosome 19 were correlated with higher expression and longer EFS in all 151 analyzed AML cases (Supplemental Table S12-S13). These two HERVK9 ORFs originated from the same HERVK9 element (Fig. 3e). Their expression was validated by reverse-transcription quantitative polymerase chain reaction (PCR) of AML-derived cell lines (Fig. 3f and Supplemental Figure S4c). The DNA sequences of the PCR fragments were confirmed by Sanger sequencing (data not shown).

### Association between higher HERVK9 expression and allogeneic immune reactions towards AML cells and apoptotic signaling

**Fig. 4:**
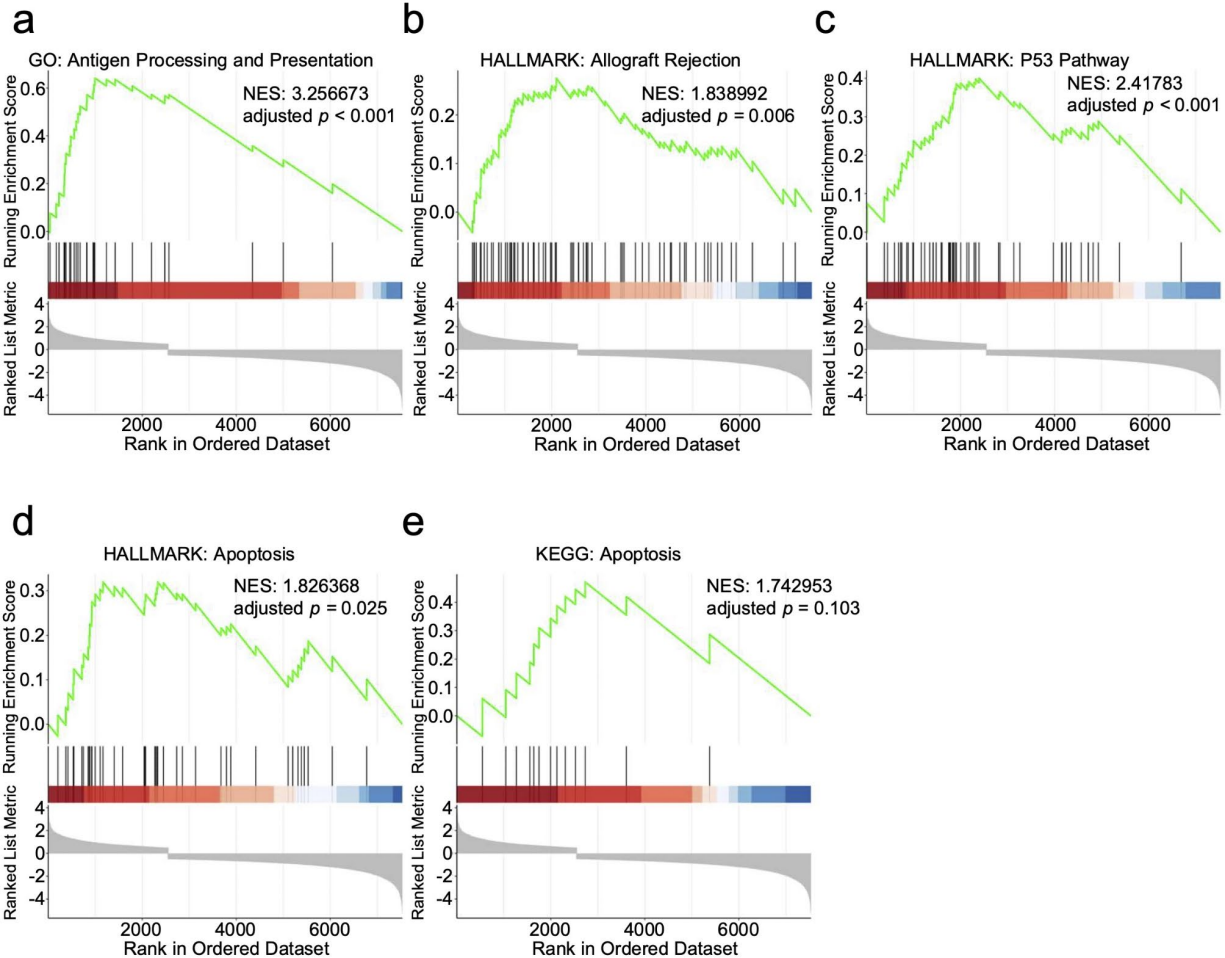
Gene set enrichment analyses (GSEAs) of differentially expressed human genes in AML cells with high HERVK9 expression. Abbreviations: NES, normalized enrichment score. Detailed information is available in Supplemental Table S14-S16

A previous study indicated that an HERV-derived peptide presented on MHC class I of renal cell carcinoma cells could induce cytotoxic T cell activation and clonal expansion, leading to an anti-neoplastic immunoreaction [25]. Using BLASTP on the gEVE website, we discovered that the original amino acid sequence of this HERV was unique to HERVK9. To further investigate whether the aberrant expression of HERVK9-derived ORFs contributes to anti-neoplastic immunity against AML, we analyzed the immunological phenotype of AML cells with higher HERVK9 expression. We performed gene set enrichment analyses (GSEAs) of differentially expressed human genes using the hallmark gene sets (Supplemental Table S14), KEGG legacy subset of canonical pathways (Supplemental Table S15), and Gene Ontology gene sets obtained from MSigDB (Supplemental Table S16). Gene sets associated with immune responses were upregulated in the AML group with higher levels of expression of HERVK9 elements (Supplemental Table S14-S16). Furthermore, gene sets related to antigen processing and presentation (Fig. 4a), allograft rejection (Fig. 4b), the p53 pathway (Fig. 4c), and apoptosis (Fig. 4d-e) were significantly upregulated in the high HERVK9 expression group. These results were validated using the RNA-seq data from AML cells in the GSE49642 dataset (Supplemental Figure S5). The hallmark gene set of allograft rejection includes the upregulated genes of allogeneic transplanted cells targeted by host immune cells, suggesting that AML cells with higher HERVK9 expression may undergo apoptosis via an alloreactive immune response mediated by aberrant antigen processing and presentation on major histocompatibility complexes. Taken together, these results suggest a potential relationship between the aberrant HERVK9 expression in AML and certain adoptive immune responses, which may contribute to improved disease control independently of allo-HCT-mediated alloreactive immune responses.

## Discussion

In this study, we investigated the correlation between the expression profiles of EVE-derived ORFs in AML cells and patient outcomes through a pan-transcriptomic analysis. We found that HERVK9-derived ORFs, especially those on chromosome 19, were associated with prolonged EFS in intensively treated AML cases. Although many previous studies have reported the aberrant expression of EVEs [39–43], little is known about their impact on patient outcomes. Our findings strongly suggest that EVE-derived ORFs are associated with prolonged EFS. Furthermore, our data suggest that AML cells with higher levels of HERVK9-derived ORF expression exhibit upregulation of genes associated with antigen presentation and responses to adaptive immune reactions. Since most EVE-derived ORFs are not expressed in normal tissues or organs, our results indicate the potential that EVE-derived peptides may be synthesized, processed, and presented in major histocompatibility complexes as cancer neoantigens. We previously reported that HERV3-1 protein was highly expressed in AML patients, particularly in the monocytic lineage, although the association of their expression and clinical profile are unclear [42]. A recent study revealed that the expression of two HERV-derived genes, Suppressyn and Syncytin-2, affects the prognosis of AML via the activation of immune cell infiltration [43]. In the present study, we systematically analyzed the expression of all EVE-derived ORFs and identified HERVK9-derived ORFs as the strongest prognostic contributors in AML. Given that neoantigens serves as ideal targets for immunotherapies, including allo-HCT and chimeric antigen receptor T-cell therapy, further studies are warranted to identify the peptides translated from HERVK9 in AML cells.

Clinically, one of the most critical aspects of AML treatment is the precise determination of the necessity for upfront allo-HCT at diagnosis. Although both the NCCN2017 and ELN2017 risk stratifications serve as reliable indices for determining the validity of allo-HCT in improving disease control, additional stratification criteria are needed to identify poor responders to chemotherapies, particularly in cases classified as good/favorable- or intermediate-risk. Based on our findings, which revealed a positive correlation between HERVK9 expression and EFS, quantifying HERVK9 expression at diagnosis may aid physicians in assessing the necessity of upfront allo-HCT. Specifically, the strong anti-neoplastic immune response (graft-versus-leukemia effect) associated with allo-HCT may enhance the prognostic value of low-immunogenic AML cases characterized by low HERVK9 expression levels.

However, this study has several limitations. Since CD34-purified HPCs comprise a heterogenous cell population, including hematopoietic stem cells, the expression profile of EVEs in each cell type should be further analyzed using single-cell transcriptome analyses. Additionally, due to the lack of information regarding the therapeutic response to initial induction chemotherapy in the TCGA-LAML original article, our research could not assess whether upfront allo-HCT improves the EFS in AML cases classified as good/favorable- or intermediate-risk. Furthermore, we must consider the presence of unexpected confounding factors that may have influenced patient prognosis, such as variations in chemotherapy dose intensity and differences in supportive care for treatment-related toxicities. Finally, although antineoplastic immune responses cannot be fully evaluated solely based on transcriptome analysis, which remains a major limitation, our results provide strong evidence supporting the clinical impact of HERVK9 expression in AML. These findings align with prior research identifying a HERVK9-derived neoantigen in renal cell carcinoma [25], reinforcing the importance of investigating EVE functions in neoplasms in future studies.

## Conclusion

While ERVs are aberrantly expressed in AML cells, HERVK9 expression is positively associated with improved EFS in cases treated with intensive chemotherapies, independent of established risk classifications, including the FAB classification and cytogenetic or genetic abnormalities.

## Electronic supplementary material

Below is the link to the electronic supplementary material.

Supplementary Material 1

Supplementary Material 2

## Data Availability

No datasets were generated or analysed during the current study.

## Abbreviations

AML: Acute Myelocytic Leukemia
allo-HCT: Allogeneic Hematopoietic Cell Transplantation
DE: Differentially-Expressed
DEG: Differentially-Expressed Gene
EFS: Event-Free Survival
ELN2017: European Leukemia Network 2017
ERV: Endogenous Retrovirus
EVE: Endogenous Viral Elements
FAB: French-American-British
GEO: Gene Expression Omnibus
GSEA: Gene-Set Enrichment Analysis
HERV: Human Endogenous Retrovirus
HERVK9: Human Endogenous Retrovirus Family K9
HPC: Hematopoietic Precursor Cells
KEGG: Kyoto Encyclopedia of Genes and Genomes
MSigDB: Molecular Signatures Database
NCCN2017: National Comprehensive Cancer Network 2017
ORF: Open Reading Frame
PCA: Principal Component Analysis
PCR: Polymerase Chain Reaction
SRA: Sequence Read Archive
TCGA: The Cancer Genome Atlas
TPM: Transcripts Per Million
UMAP: Uniform Manifold Approximation and Projection
