# Supplementary material for: Aberrant expression of human endogenous retrovirus K9-derived elements is associated with better clinical outcome of acute myelocytic leukemia
Source: Retrovirology. 2025 Apr 1;22:4. doi: 10.1186/s12977-025-00661-6 (PMC11959769; doi:10.1186/s12977-025-00661-6)
Supplement: Supplementary file 1 — Supplementary Material 1 [file 12977_2025_661_MOESM1_ESM.docx]

**Supplemental Figures**

**
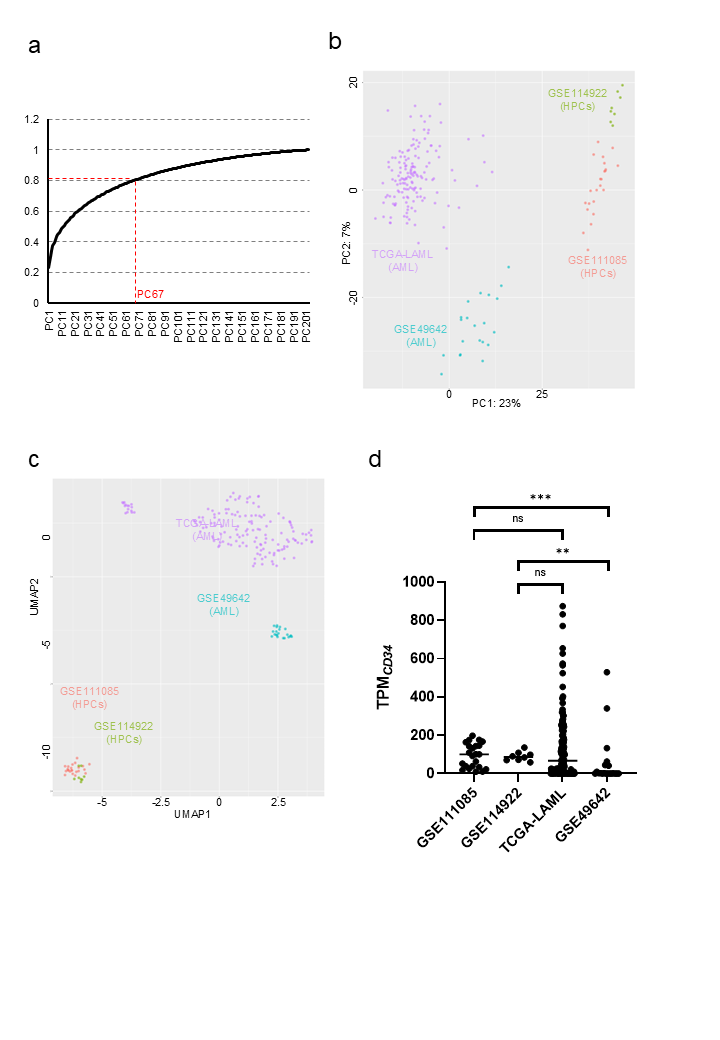
**

**Supplemental Figure S1**. Collection of analyzed datasets and check the batch effects and sample qualities

**a.** Cumulative proportion of PC. Cumulative proportion exceeds 80% at PC67 (red line; 80.059%).

**b-c.** Dot plot of PC1 and PC2 (b) and two-dimensional UMAP of PC1 to PC67. Four collected datasets were annotated by color.

**d.** Transcripts per million (TPM) values of all analyzed samples. Mann-Whitney’s U test was used for statistical analyses, and results were indicated as follows; “ns” with p >0.05, “**” with p <0.01, and “***” with p <0.001.


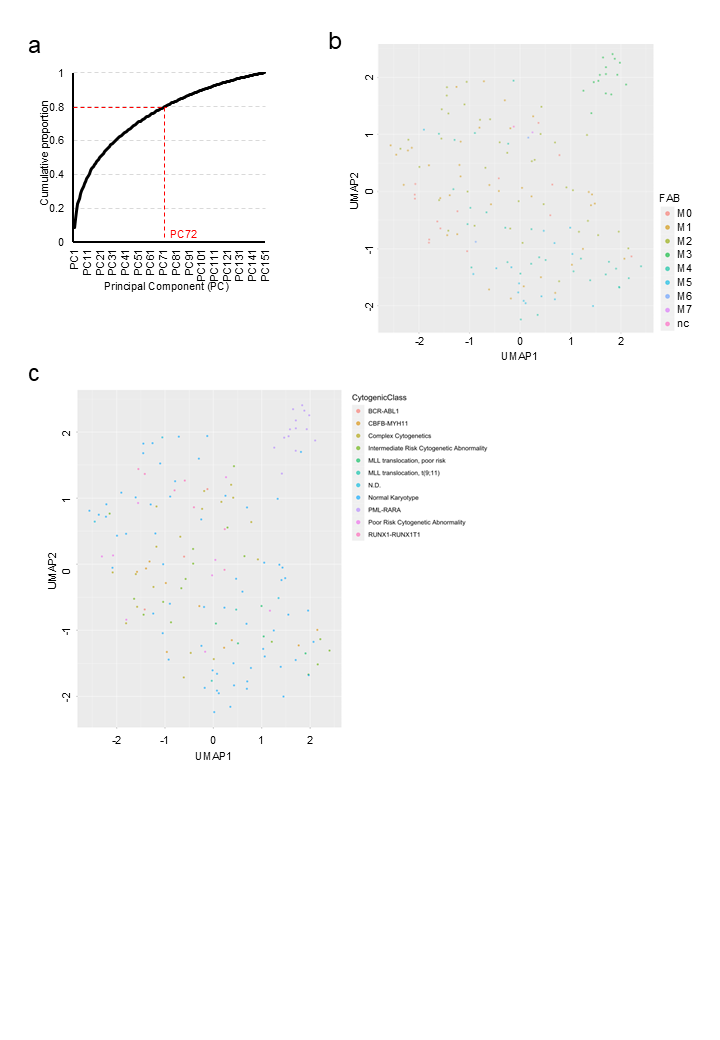


**Supplemental Figure S2. Unsupervised clustering of expression pattern of 1517 DE-EVE ORFs in AMLs from TCGA-LAML.**

**a.** Cumulative proportion of PC. Cumulative proportion exceeds 80% at PC72 (red line; 80.025%).

**b-c.** Two-dimensional UMAP of PC1 to PC72. Cytogenetic abnormalities (**b**) and FAB morphological classification (**c**) of analyzed AMLs were annotated by color.


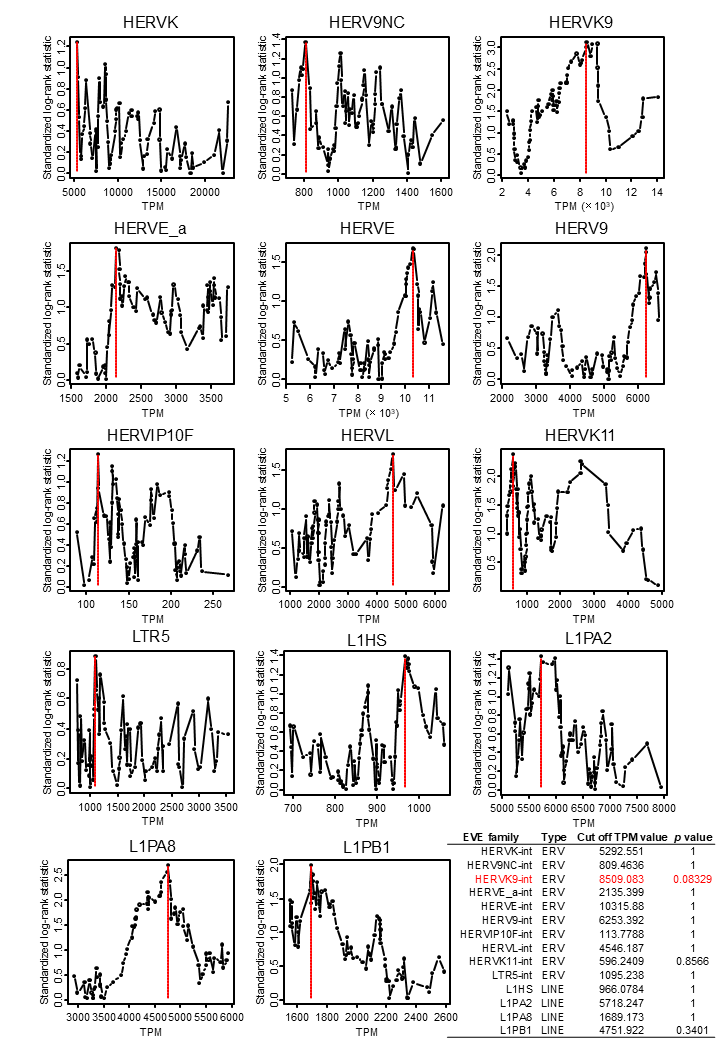


**Supplemental Figure S3. Maximally selected rank statistics of 14 detected DE-EVE ORFs in 90 TCGA-LAML cases.**

Hothorn and Lausen’s method was applied for approximation of *p* values. TPM, transcripts per million.


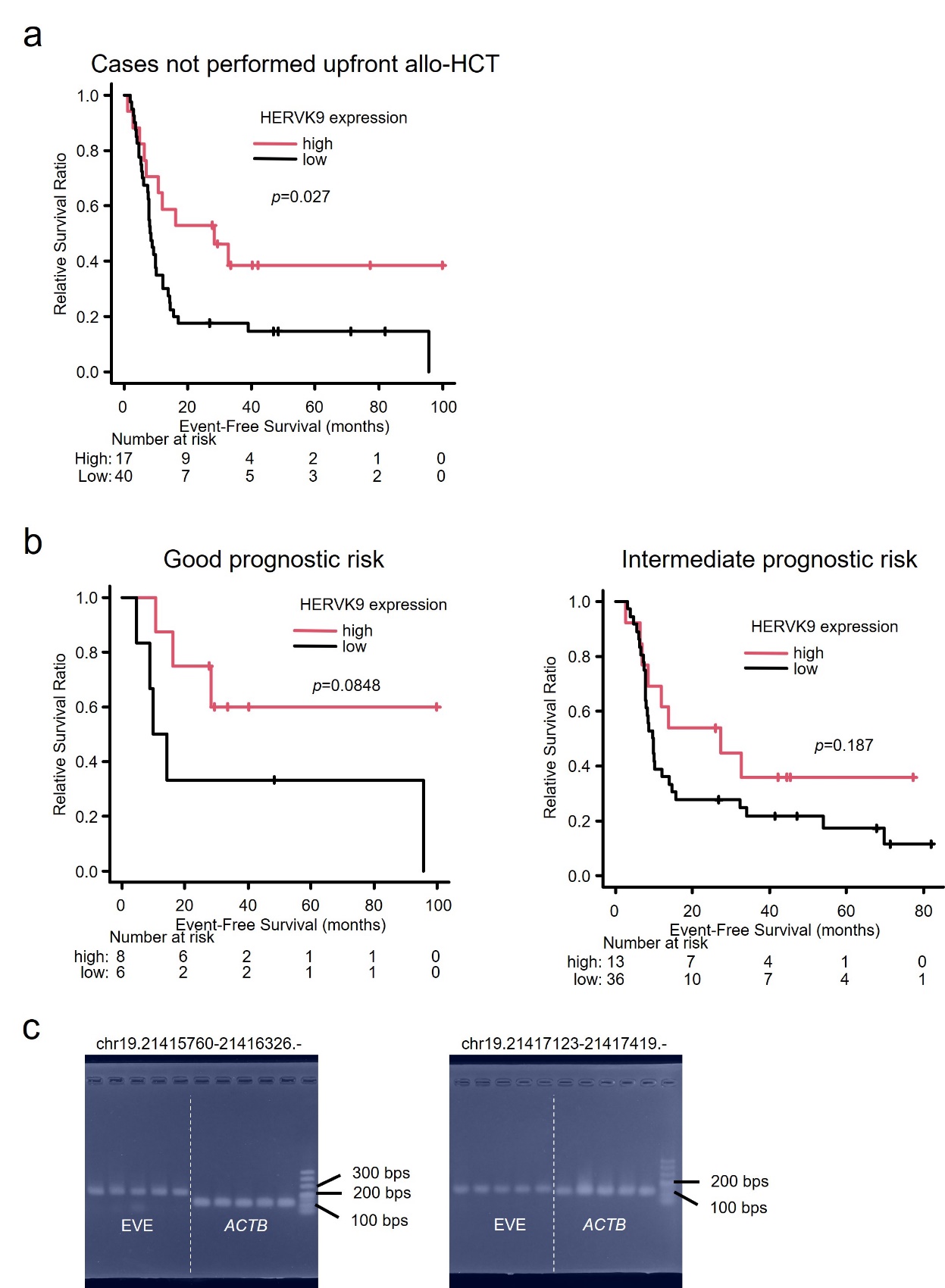


**Supplemental Figure S4. HERVK9 expression value is associated with AML prognosis**

**a.** Overall survival curves of 90 AML cases grouped according to HERVK9 expression value. Cut-off TPM value of was as same as that calculated in **Figure 3b**.

**b.** EFS cure of AML cases classified as good- and intermediate-risk according to NCCN2017.

**c.** Noncropped gel pictures of **Figure 3f**.


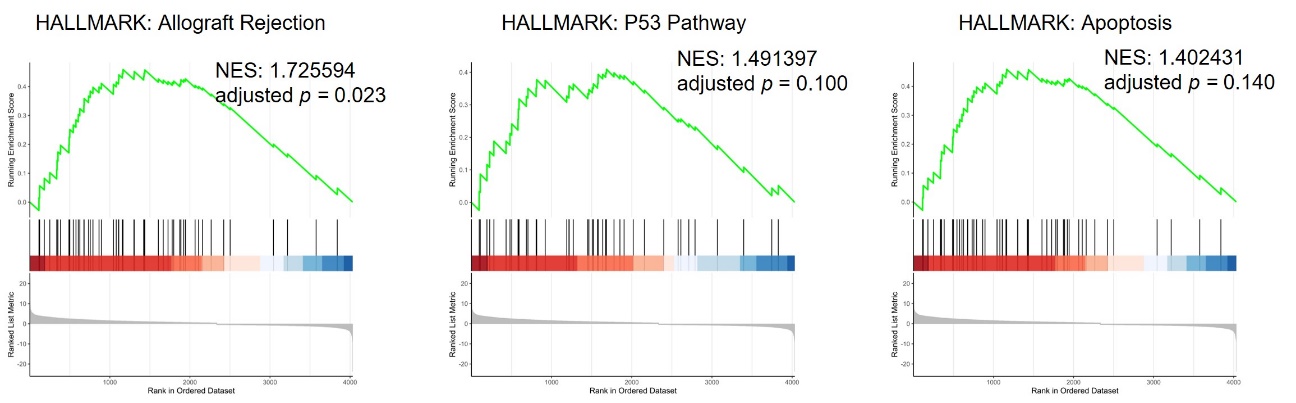


**Supplemental Figure S5. GSEAs of DE human genes in AML cells with higher HERVK9 expression in GSE49642.**

Cut-off HERVK9 expression value (TPM) was 8509.083. Abbreviation: NES, normalized enrichment score.

**Supplementary Tables**

**Supplemental Table S1.** RNA-seq data of patient-derived AMLs and hematopoietic stem cells used in this study

**Supplemental Table S2.** Primer sequences used for quantitative polymerase chain reactions.

**Supplemental Table S3.** DE-EVE ORFs of AMLs in TCGA-LAML compared to HPCs in GSE111085

**Supplemental Table S4.** DE-EVE ORFs of AMLs in TCGA-LAML compared to HPCs in GSE114922

**Supplemental Table S5.** DE-EVE ORFs of AMLs in GSE49642 compared to HPCs in GSE111085

**Supplemental Table S6.** Extracted 1,796 commonly extracted DE-EVEs

**Supplemental Table S7.** GSEA of AMLs in TCGA-LAML and HPCs in GSE111085

**Supplemental Table S8.** GSEA of AMLs in GSE49642 and HPCs in GSE111085

**Supplemental Table S9.** Common enriched ERV families and core enriched EVE ORFs

**Supplemental Table S10.** Clinical information and expression status of HERVK9 of 151 AML cases in TCGA-LAML

**Supplemental Table S11.** Association of HERVK9 expression status and cytogenetic abnormalities or gene mutations contributing to risk stratification of AML

**Supplemental Table S12.** Survival analyses of 151 AML cases grouped by expression values of HERVK9-derived ORFs.

**Supplemental Table S13.** TPM values of 21 commonly enriched HERVK9 elements in AMLs of TCGA-LAML

**Supplemental Table S14.** GSEA of DE human genes using hallmark gene sets from MSigDB

**Supplemental Table S15.** GSEA of DE human genes using KEGG pathway database from MSigDB

**Supplemental Table S16.** GSEA of DE human genes using data sets of GO from MSigDB
